# Supplementary material for: Architecture of Nanoantioxidant Based on Mesoporous Organosilica Trp-Met-PMO with Dipeptide Skeleton
Source: Materials (Basel). 2023 Jan 9;16(2):638. doi: 10.3390/ma16020638 (PMC9863312; doi:10.3390/ma16020638)
Supplement: Supplementary file 1 [file materials-16-00638-s001.zip › materials-2064778-supplementary.pdf]

Architecture of Nanoantioxidant Based on Mesoporous Organosilica Trp-Met-PMO with Dipeptide Skeleton

Wanli Zhou, Haohua Ma, Yunqiao Dai, Yijing Du, Cheng Guo, Jianqiang Wang*

School of Chemistry and Molecular Engineering, Nanjing Tech University, 30 Puzhu South Road, Jiangsu, Nanjing, 211816, China

E-mail: jqwang@njtech.edu.cn

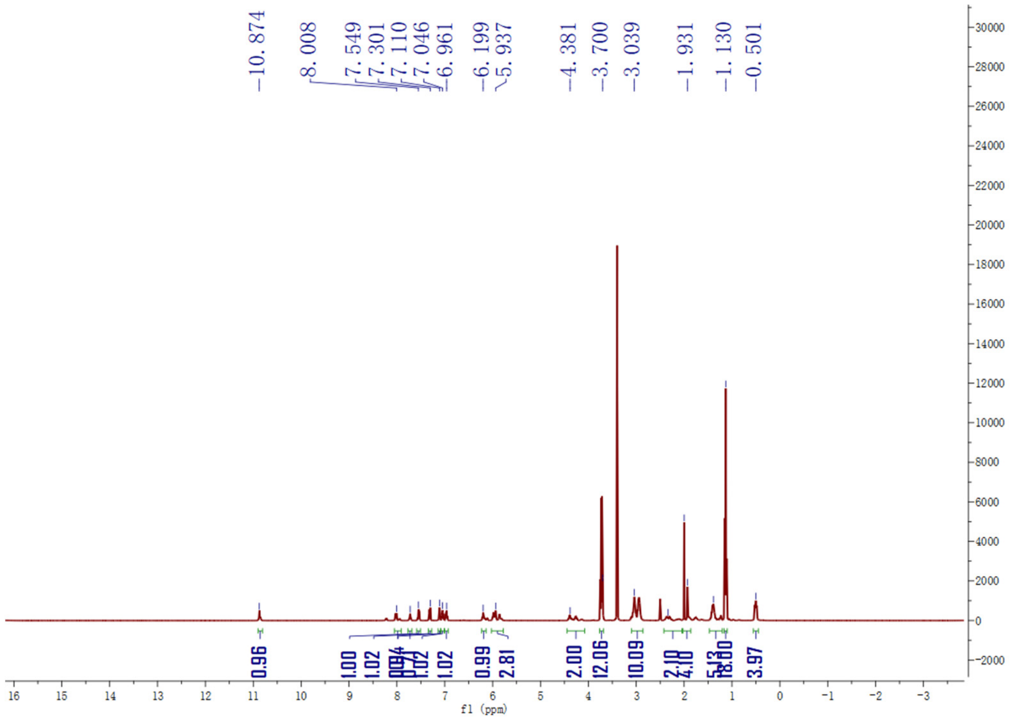

Figure S1. 1H NMR of organosilica precursor Trp-Met-Si

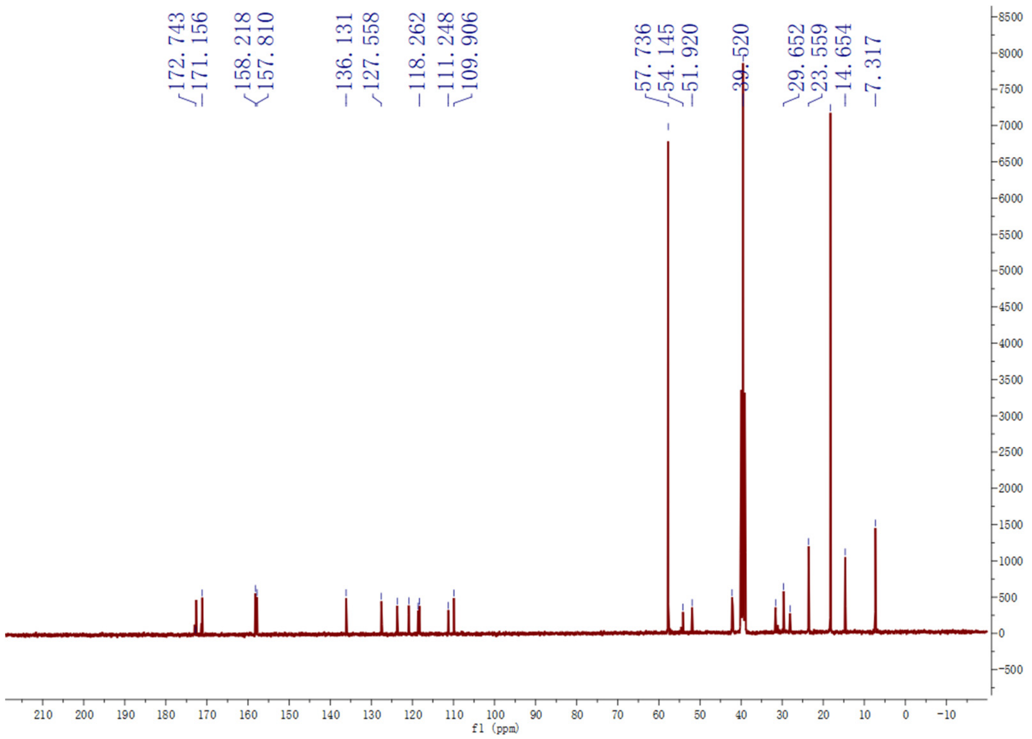

Figure S2. 13C NMR of organosilica precursor Trp-Met-Si

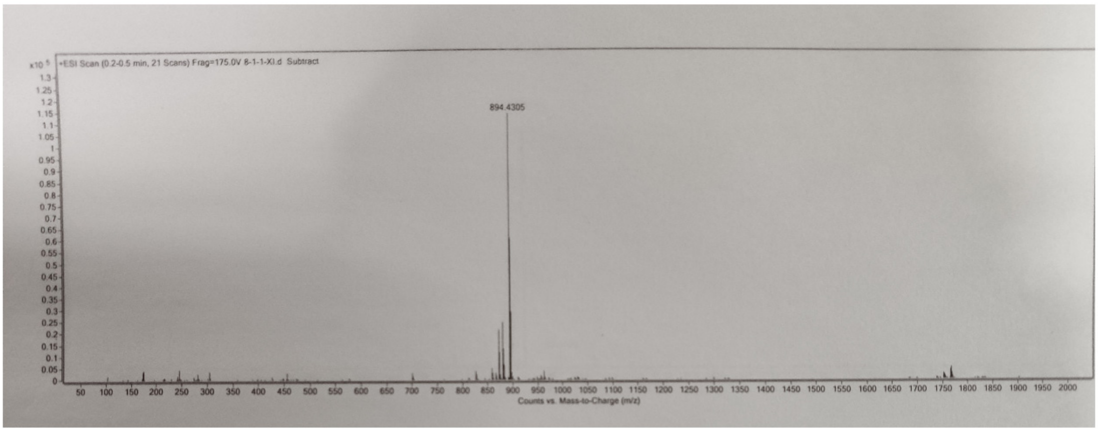

Figure S3. ESI-MS of organosilica precursor Trp-Met-Si

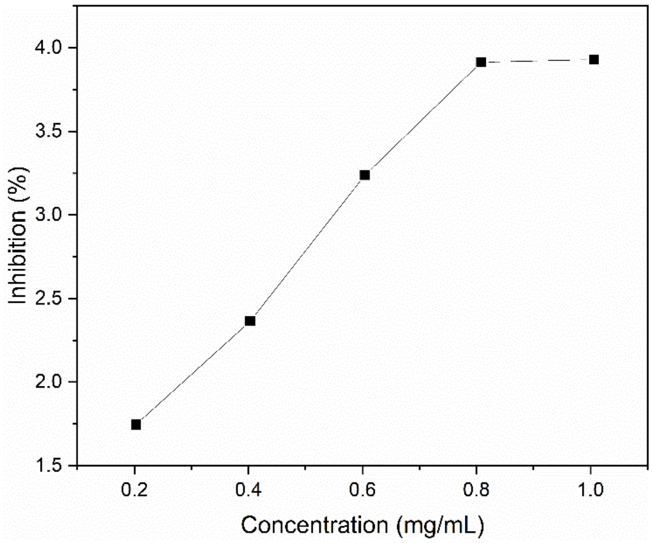

Figure S4. Scavenging effect of different concentrations of Trp-Met-20-PMO on ABTS free radicals
